# Elevated Expression of miR-296 in Human Placentas and Serum Samples From Pregnancies With Preeclampsia

**DOI:** 10.3389/bjbs.2023.11004

**Published:** 2023-04-11

**Authors:** Dandan Zhu, Ting Guo, Jie Xu, Donglan Yuan, Mei Lin, Minyan Yang

**Affiliations:** ^1^ The Department of Obstetrics and Gynecology, Taizhou People’s Hospital Affiliated to Nanjing University of Traditional Chinese Medicine, Taizhou, China; ^2^ Institute of Clinical Medicine, Taizhou People’s Hospital Affiliated to Nanjing University of Traditional Chinese Medicine, Taizhou, China; ^3^ Department of Clinical Laboratory, Taizhou People’s Hospital Affiliated to Nanjing University of Traditional Chinese Medicine, Taizhou, China

**Keywords:** preeclampsia, miR-296, ROC analysis, early onset, late onset

## Abstract

**Background:** Preeclampsia (PE) is a hypertensive disorder of pregnancy characterized by widespread maternal endothelial dysfunction. Although clinical signs subside following delivery, long-term risks associated with PE include hypertension, stroke, and cardiovascular disease. MicroRNAs (miRNAs) are emerging as critical regulators of biological function, and while alterations to the miRNAs have been described in the context of pregnancy and PE, the postpartum implications of PE on miRNA expression are unknown. In the present study, we aimed to determine the clinical performance of miR-296 in PE.

**Methods:** First, the clinical information and outcomes of all the participants were collected and analyzed. Afterward, the miR-296 expressions in the serum samples from healthy pregnant women and women with PE at different periods were detected using quantitative real-time polymerase chain reaction (qRT-PCR). Then, the receive operation characteristic (ROC) curve was used to determine the diagnostic value of miR-296 in PE. Finally, the at-term placentals were collected, the expressions of miR-296 in different groups were compared at first blood collection and at delivery.

**Results:** In this study, we found that miR-296 expression was significantly increased in the placenta samples from PE patients compared with that in healthy controls both in early onset group (EOPE, *p* < 0.01) and late onset group (LOPE, *p* < 0.01). Furthermore, results of ROC analysis showed miR-296 might be a putative biomarker for early onset preeclampsia and late onset preeclampsia diagnosis with an area under the curve (AUC) of 0.84 (95% confidence interval 0.75–0.92) and 0.85 (95% confidence interval 0.77–0.93). Last but not the least, the expressions of miR-296 were significantly increased (*p* < 0.05) in serum samples of EOPE and LOPE patients (*p* < 0.001), and serum and placental levels of the miR-296 was positively correlated for EOPE (*r* = 0.5574, *p* < 0.001) and LOPE (*r* = 0.6613, *p* < 0.001) patients, respectively. Meanwhile, compared with those at first blood collection, the expression of miR-296 in EOPE (*p* = 0.05) and LOPE (*p* = 0.01) were significantly decreased at delivery.

**Conclusion:** miR-296 may function as a putative diagnostic biomarker for PE and contribute to identifying at-risk mothers in pregnancy.

## Introduction

Preeclampsia (PE) is a leading cause of maternal and neonatal mortality and morbidity around the world. However, the pathophysiology of this disease is not yet fully elucidated (1). Although the cause and consequence relationship is unclear, familial and epidemiological studies have shown a complex interactions between genetics and environmental factors and the outcome of complications of PE birth (2, 3). In addition, infection and inflammatory pathologies have been proved to cause and enhance PE (4, 5). Hence, it’s urgent to find an easy way to identify those at-risk mothers in pregnancy.

miRNAs are a group of small, non-coding RNAs (∼22 nucleotides in length) which could regulate gene expression by degradation or restraining translation of messenger RNA transcriptions (6). In spite of the biological significance of a variety of miRNAs remains to be further investigated, functional studies have implicated their regulatory actions on different developmental processes, and their aberrant expression is associated with developmental abnormalities, cell cycle development and apoptosis, inflammation and immune regulation, as well as cellular transformation and cancer. To our best of our knowledge, abnormal miRNA expression has been confirmed in various diseases, including diabetes and PE (7, 8). Recently, a growing number of studies have reported the expression of a large number of miRNAs in placentas and fetal membranes with aberrant expression in these tissues under the influence by PE (9–11). A previous study pointed out that miR-296 was upregulated in PE patients (12). However, the relationship between miR-296 and PE and its potential clinical value remain unclear.

In the present study, we determine the expression level of miR-296 in PE patients. Additionally, we test the clinical roles of miR-296 in PE in order to contribute to clinical diagnosis and treatment. We hypothesized that miR-296 is upregulated in both early onset PE (EOPE) and late onset PE (LOPE) patients, and may function as potential biomarkers.

## Methods

### Patient Samples

200 serum samples and placentas from pregnancies were obtained from the Taizhou People’s Hospital including 50 early onset preeclampsia pregnancies, 50 pregnancies at 20–33 gestational weeks, 50 late onset preeclampsia pregnanciesa and 50 pregnancies at 34–41 gestational weeks collected in red tiger-top gel separator tubes (Thermo Fisher Scientific, Shanghai, China). Preeclampsia was defined as hypertension—systolic blood pressure ≥140 mmHg or diastolic blood pressure ≥90 mmHg, on at least 2 occasions, at least 4 h apart. Patients with diabetes mellitus, PE premature ruptured membranes without labor, multiple gestations, and fetal demise *in utero* or fetal anomalies were excluded from this study.

Immediately after delivery, the overlaying fetal membranes were removed and several small pieces of placental tissues were randomly collected, blotted on clean tissue paper to remove excess blood and placed in cryotubes containing RNAlater (Qiagen Inc., Valencia, California), at 4 to 1 volume of tissue.

Participants fasted overnight prior to tests under standardized conditions. Fasting blood samples were taken from participants and collected. After separated by centrifugation at 10,000 x g and kept at −80°C until use. Clinicopathological features in 200 patients were displayed in Table 1 and the outcomes of 200 patients were displayed in Table 2.

**TABLE 1:** Clinicopathological features of participants.

Features	20–33 control (*n* = 50)	EOPE (*n* = 50)	p value
Age (years)	26.8 ± 6.9	27.5 ± 5.7	0.5815
BMI (kg/m^2^)	22.1 ± 1.3	22.0 ± 1.5	0.7224
GW at admission	28.1 ± 3.2	27.9 ± 2.7	0.7363
SBP (mmHg)	107.5 ± 9.8	158.6 ± 11.1	<0.001
DBP (mmHg)	72.3 ± 5.8	106.8 ± 10.1	<0.001
Nulliparity (%)	31 (62%)	25 (50%)	0.0874
**Features**	**34–41 control (*n* = 50)**	**LOPE (*n* = 50)**	**p value**
Age (years)	31.7 ± 6.9	29.8 ± 7.2	0.1810
BMI (kg/m^2^)	21.9 ± 1.6	22.2 ± 1.7	0.3658
GW at admission	38.1 ± 1.9	37.6 ± 2.2	0.2268
SBP (mmHg)	104.3 ± 8.7	152.9 ± 9.6	<0.001
DBP (mmHg)	73.5 ± 6.9	102.4 ± 7.0	<0.001
Nulliparity (%)	24 (48%)	27 (54%)	0.3960

BMI, body mass index; GW, gestational weeks; 20–33 control, healthy pregnancies at 20–33 gestational weeks; EOPE, early onset preeclampsia; 34–41 control, healthy pregnancies at 34–41 gestational weeks; LOPE, late onset preeclampsia; SBP, systolic blood pressure; DBP, diastolic blood pressure.

**TABLE 2:** Clinical outcomes of participants.

Features	20–33 control (*n* = 50)	EOPE (*n* = 50)	p value
GW at delivery	38.8 ± 1.0	31.2 ± 3.3	<0.001
Delivery mode			0.0051
Cesarean	27	41	
Vaginal delivery	23	9	
Newborn weight (gram)	3465.0 ± 371.3	1012.7 ± 415.6	<0.001
Newborn height (cm)	48.9 ± 0.3	33.2 ± 0.2	<0.001
Newborn sex (male/female %)	0.8519	0.7857	0.9254
Placental weight (gram)	540.6 ± 69.7	168.1 ± 75.8	<0.001
**Features**	**20–33 control (*n* = 50)**	**EOPE (*n* = 50)**	**p value**
GW at delivery	39.2 ± 1.1	38.1 ± 0.8	<0.001
Delivery mode			0.0003
Cesarean	20	38	
Vaginal delivery	30	12	
Newborn weight (gram)	3390.1 ± 385.6	2696.4 ± 329.4	<0.001
Newborn height (cm)	49.2 ± 0.2	41.5 ± 0.3	<0.001
Newborn sex (male/female %)	0.6129	0.7241	0.8780
Placental weight (gram)	571.1 ± 63.8	425.0 ± 54.9	<0.001

GW, gestational weeks; 20–33 control, healthy pregnancies at 20–33 gestational weeks; EOPE, early onset preeclampsia; 34–41 control, healthy pregnancies at 34–41 gestational weeks; LOPE, late onset preeclampsia; SBP, systolic blood pressure; DBP, diastolic blood pressure.

### Reverse-Transcription Quantitative Real-Time PCR (RT-qPCR) Assay

Total RNA was extracted from 500 μL serum samples and placentas using TRIzol LS reagent (Life Technologies, Paisley, United Kingdom) under the instructions. RNA samples were quantified in a NanoDrop One spectrophotometer (Thermo Scientific, United States) and quality/integrity was checked in a 2100 Bioanalyzer (Agilent, United States). 2 μL cDNA was synthesized using the TaqMan MicroRNA Reverse Transcription kit (Applied Biosystems, Foster City, CA, United States). Then, the expression of miR-296 was detected by SYBR Green PCR kit (Qiagen, Valencia, CA, United States) according to the manufacturer’s protocol. The thermal cycling conditions were as follows: 94°C for 3 min, 40 cycles of 94°C for 5 s, 65°C for 20 s, final extension at 65°C for 5 min. U6 functioned as the internal control. The relative expression of miR-296 was normalized to the level of U6 small nuclear RNA using the 2^−△△Ct^ method. The sequences were as follows: miR-296 forward, 5′-GGA​GTG​TAG​GCC​CAA​TAC​CAG​A-3′; and reverse 5′-TGC​CAC​TTA​GCA​GCA​CAG​AAA-3′; U6 forward, 5′-AGA​GCC​TGT​GGT​GTC​C-3′; and reverse 5′-CAT​CTT​CAA​AGC​ACT​TCC​CT-3′.

### Statistical Analysis

All data were performed using the SPSS version 17.0 statistical software and presented as mean ± standard deviation (SD). ROC curve was constructed to determine the clinical diagnostic value of miR-296 in PE. Student’s t-test and one-way ANOVA followed by the *post hoc* Turkey’s test was used to distinguish differences among groups. P value of less than 0.05 was considered to be statistically significant. Power analysis has been conducted using by GPower software (version3.1.9.7), actual power is greater than 0.8 when the samples size was 50 therefore the sample size has been set as *n* = 50.

## Results

### Clinicopathological Features of Participants

The clinical features of all the participants are summarized in Table 1. From the results, there were no significant differences of average age, body mass index (BMI), gestational weeks (GW) at first blood collection and nulliparity between 20–33 gestational weeks control and EOPE or 34–41 gestational weeks control and LOPE (Table 1, *p* > 0.05). However, the significant elevated blood pressure was detected in EOPE and LOPE as compared with 20–33 gestational weeks and 34–41 gestational weeks respectively (*p* < 0.01).

### Clinical Outcomes of Normal Pregnancy and PE

As shown in Table 2, the clinical outcomes of 20–33 GW control, EOPE, 34–41 GW control and LOPE groups were analyzed. The GW at delivery, delivery mode, newborn weight, newborn height and placental weight presented significant differences between 20–34 GW control and EOPE or 34–41 GW control and LOPE, while there were no significant differences in newborn sex.

### Expressions of miR-296 in PE and Healthy Pregnancies

**FIGURE 1:**
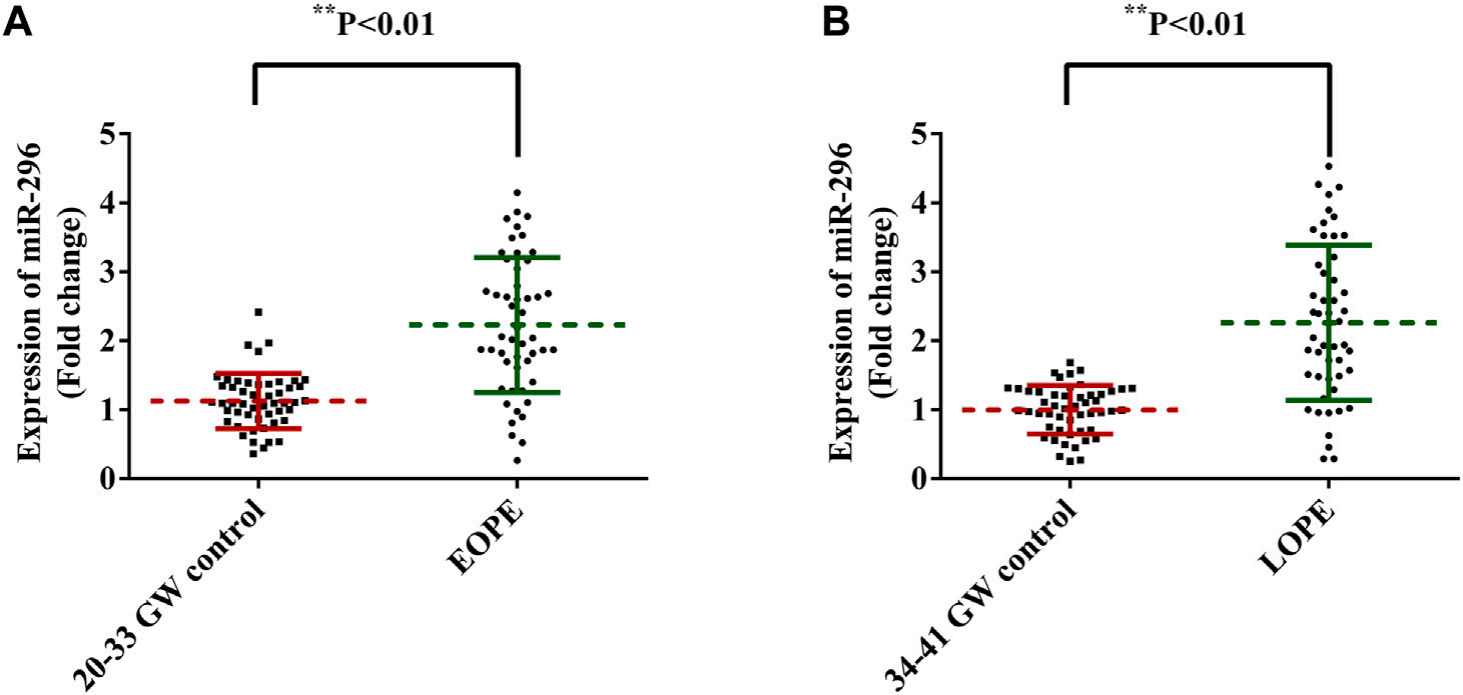
miR-296 expression between PE and control groups. miR-296 expressions in serum samples from PE and healthy pregnancies were measured by qRT-PCR analysis. **(A)** The expression level of miR-296 in serum samples from EOPE and healthy pregnancies at 20–33 gestational weeks. **(B)** The expression level of miR-296 in serum samples from LOPE and healthy controls at 34–41 gestational weeks. *p* < 0.01. PE, preeclampsia; GW, gestational weeks; EOPE, early onset preeclampsia; LOPE, late onset preeclampsia.

qRT-PCR analysis was employed to analyze placenta miR-296 expression levels in four different groups: 20–33 GW control (*n* = 50), EOPE (*n* = 50), 33–41 GW control (*n* = 50) and LOPE (*n* = 50). As shown in Figure 1A, placenta miR-296 levels were remarkably elevated in EOPO group compared with 20–33 GW group (1.09 ± 0.06 vs. 2.23 ± 0.14, *p* < 0.001). Meanwhile, in Figure 1B the miR-296 expression was remarkably increased at LOPE group (2.26 ± 0.16) as compared with 34–41 GW control group (1.00 ± 0.05).

### Diagnosis Value of Placenta miR-296 in EOPE and LOPE

**FIGURE 2:**
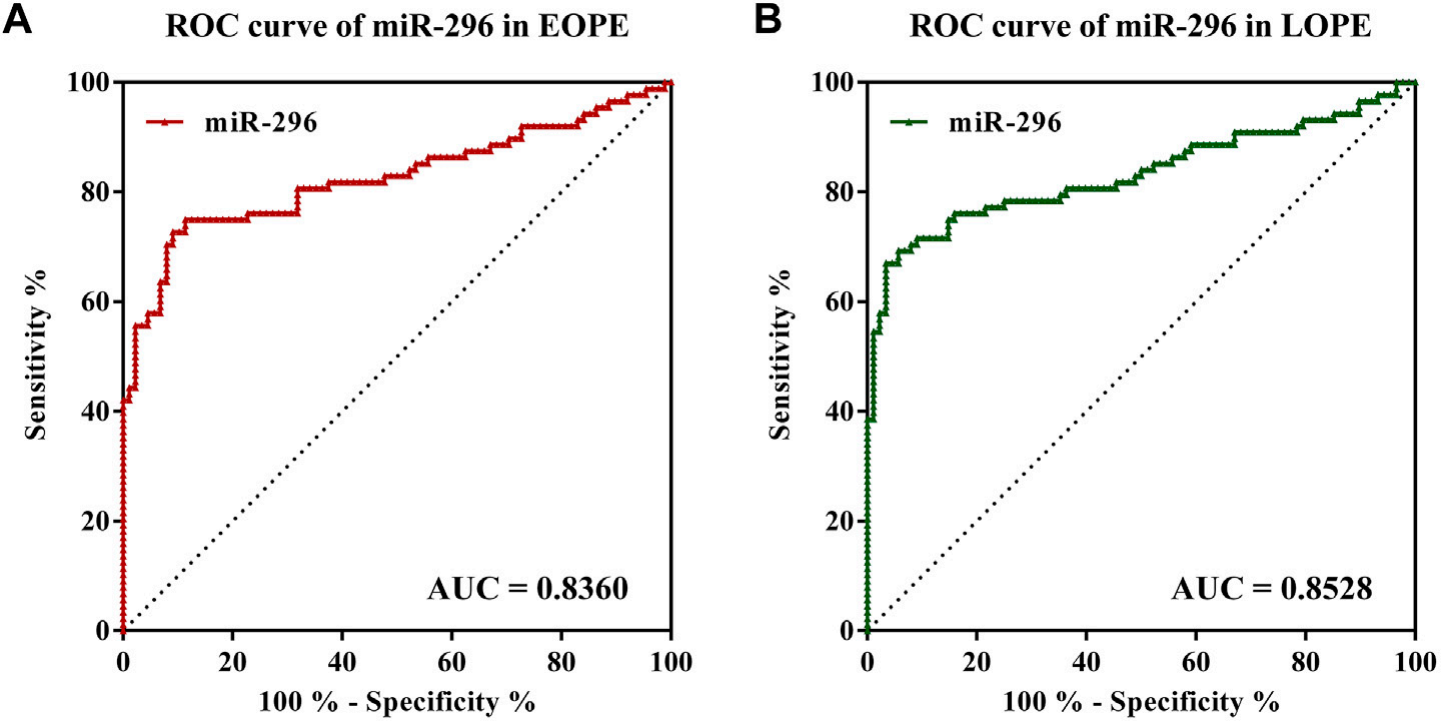
Receiver operating characteristics (ROC) curves of miR-296 discriminating healthy pregnancies from PE patients. **(A)** miR-296 manifested an area under the curve (AUC) value of 0.8360 which was greater than 0.8 and *p* < 0.05, suggesting miR-296 maybe a potential diagnostic candidate for EOPE. **(B)** miR-296 manifested an area under the curve (AUC) value of 0.8528 which was greater than 0.8 and *p* < 0.05, suggesting miR-296 maybe a potential diagnostic candidate for LOPE. PE, preeclampsia; GW, gestational weeks; EOPE, early onset preeclampsia; LOPE, late onset preeclampsia.

To find out whether placenta miR-296 could function as potential diagnostic markers for both EOPE and LOPE, ROC curves and areas under the ROC curves (AUC) were conducted and analyzed. The results showed that the expression levels of miR-296 could differentiate EOPE patients from 20–33 GW healthy pregnancies, with an AUC of 0.84 (95% confidence interval [CI] = 0.75–0.92, *p* < 0.001) (Figure 2A). The cut-off value was 1.555, positive predictive value (PPV) was 90.48% and negative predictive value (NPV) was 79.31%, likelihood ratio 9.5. Meanwhile, the ROC curves also discriminated the LOPE patients from the 34–41 GW pregnancies, with an AUC of 0.85 (95% CI = 0.77–0.93, *p* < 0.01) (Figure 2B). The cut-off value was 1.475, PPV was 88.37%, NPV was 78.95%, likelihood ratio 9.5.

### Variation of miR-296 Expressions in Serum Samples Before and at Delivery

**FIGURE 3:**
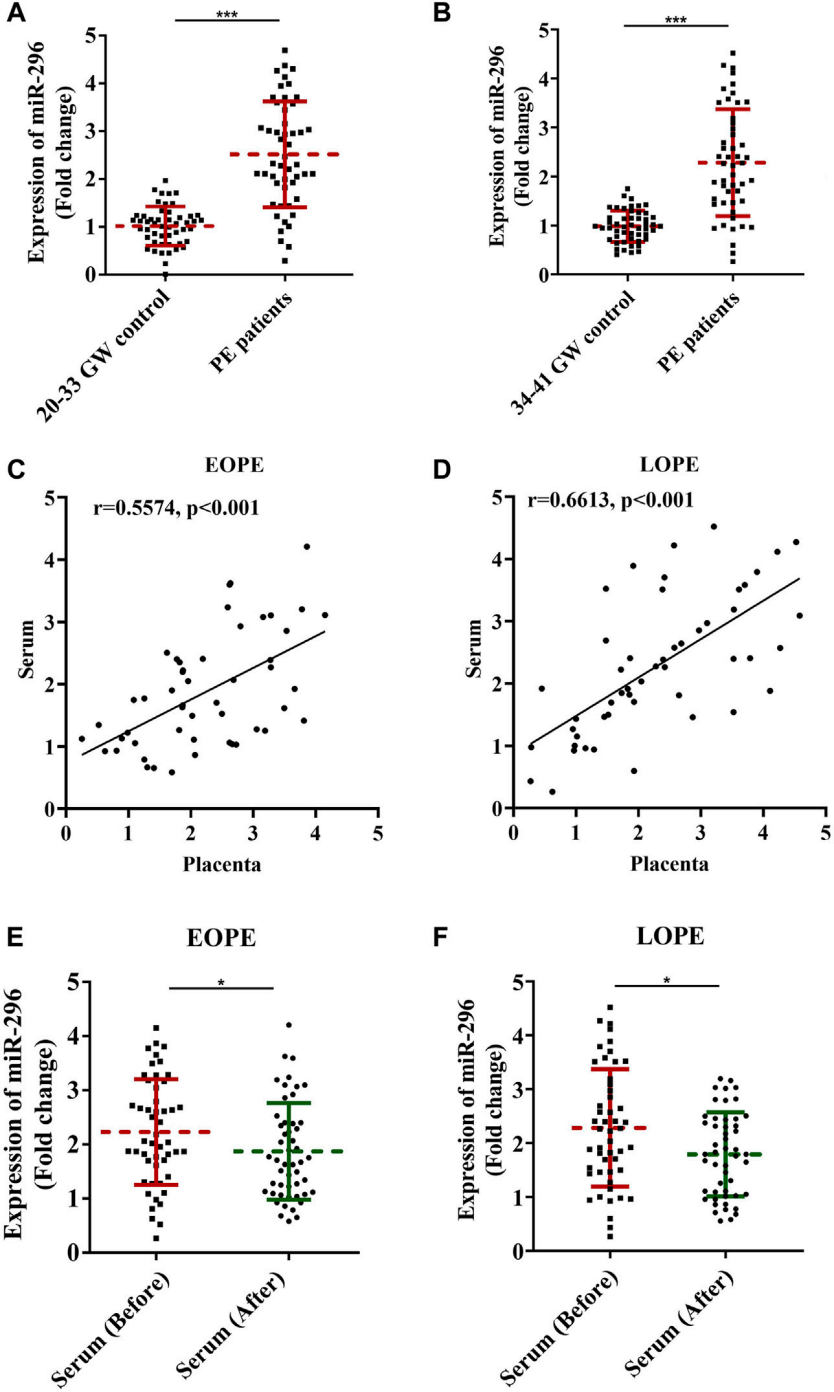
Variation of miR-296 expressions before treatment and at delivery. miR-296 expressions in different periods of pregnancy among healthy pregnancies and PE patients were compared and analyzed. **(A)** Expression of miR-296 in serum of EOPE patients and healthy controls. **(B)** Expression of miR-296 in serum of LOPE patients and healthy controls. **(C)** Results of correlation analysis for serum and placenta expression of miR-296 in EOPE patients. **(D)** Results of correlation analysis for serum and placenta expression of miR-296 in LOPE patients. **(E)** Expression of miR-296 in serum of EOPE patients at first blood collection and at delivery. **(F)** Expression of miR-296 in serum of LOPE patients at first blood collection and at delivery. EOPE, early onset preeclampsia; LOPE, late onset preeclampsia. *, *p* < 0.05, ***, *p* < 0.001.

In order to verify whether miR-296 could be a biomarker for treatment response in PE, we compared the expressions of miR-296 in serum samples from all pregnancies and placentas at first blood collection and at delivery. As shown in the results, at delivery, the expressions of miR-296 were significantly increased in serum samples from EOPE and LOPE patients as compared with time-matched control groups (Figures 3A,B, *p* < 0.01). Furthermore, results of correlation analysis showed that serum and placental levels of the miR-296 were positively correlated for EOPE (Figure 3C, *r* = 0.5574, *p* < 0.001) and LOPE (Figure 3D, *r* = 0.6613, *p* < 0.001) patients, respectively. What was more, compared with those at first blood collection, the expression of miR-296 in EOPE (Figure 3E, *p* = 0.05) and LOPE (Figure 3F, *p* = 0.01) were significantly decreased at delivery.

## Discussion

In our present study, we have identified that placenta miR-296 levels were significantly increased both in EOPE and LOPE when compared to gestational weeks matched normal pregnancy. Furthermore, this is the first report suggesting a potential role of miR-296 in the diagnosis of EOPE and LOPE, contributing to clinical diagnosis and treatment.

miR-296 is a family of microRNA precursors abundantly found in mammals, including humans. In fact, miR-296 has been named as “angiomiR” since it was proved to regulate angiogenesis and the creation of new blood vessels (13, 14). Specially, in the realm of cancer, miR-296 was thought to have a crucial roles in promoting tumor angiogenesis. For instance, miR-296 was considered to exert pro-angiogenic function in glioma and malignant melanoma (15, 16). Interestingly, we found that the miR-296 expressions in the serum samples from normal pregnancies (20–33 gestational weeks and 34–41 gestational weeks) were significantly decreased when compared to EOPE or LOPE patients. Murphy (17) proposed that miR-296 was abundantly expressed in maternal placenta in severe PE patients compared with time-matched healthy pregnancies. Mayor-Lynn et al (18) also reported that miR-296 expression was markedly elevated in placentas from spontaneous preterm delivery.

The expressions of miRNAs maybe changed during the clinical treatment, which may indicate the treatment response of certain diseases. In rectal cancer, miR-21, miR-99b and miR-375 combination could be used as promising response biomarker for chemoradiotherapy (19). Piatopoulou et al (20) also suggested that miR-125b may be a candidate for poor response after acute lymphoblastic leukemia chemotherapy treatment. In the current study, we further investigated the potential role of miR-296 in diagnosing PE and predicting PE outcome. The outcome data of the participants revealed that there were significant differences among PE and healthy pregnancies such as gestational weeks at delivery, delivery mode, newborn weight, newborn height and placentas weight. Meanwhile, our results indicated that the expressions of miR-296 were significantly upregulated in serum samples and placentas from EOPE and LOPE while slightly decreased at delivery compared with those at first collection, and the serum and placental levels of the miR-296 were positively correlated. More importantly, the ROC analysis revealed that the AUC of miR-296 in discriminating EOPE from time-matched pregnancies was 0.86 with the 95% CI = 0.75–0.92 (*p* < 0.01) and miR-296 may also differentiate LOPE patients from healthy pregnancies at the matched time with the AUC of 0.85 (95% CI = 0.77–0.93).

Considering that miR-296 expression was upregulated in the serum samples and placentas of EOPE and LOPE patients at different periods compared with healthy controls, examining their levels in the enrolled pregnant women could serve as a promising indicator for PE risk. However, these observations should be further investigated among a larger number of samples designed by a multi-center study.

## Conclusion

Expressions of miR-296 in serum samples and placentas from healthy pregnancies and PE patients with different periods of pregnancy were determined in this study. For the first time, we revealed the clinical value of miR-296 as a promising biomarker for PE diagnosis. In light of the results, we could conclude that miR-296 maybe a novel target for discriminating both early onset PE and late onset PE from healthy pregnancies.

## Summary Table

### What is Known About This Topic

• In this study, we found that miR-296 expression was significantly increased in the serum samples from PE patients compared with that in healthy controls both in early onset group and late onset group.
• Furthermore, miR-296 might be a putative biomarker for early onset preeclampsia and early onset preeclampsia diagnosis with an area under the curve (AUC) of 0.8360 and 0.8528.
• Last but not the least, the expressions of miR-296 were significantly increased in serum samples and placentas from EOPE and LOPE while slightly decreased at delivery compared with those at first collection.

### What This Work Adds

• Expressions of miR-296 in serum samples and placentas from healthy pregnancies and PE patients with different periods of pregnancy were determined in this study.
• For the first time, we revealed the clinical value of miR-296 as a promising biomarker for PE diagnosis.
• In light of the results, we could conclude that miR-296 maybe a novel target for discriminating both early onset PE and late onset PE from healthy pregnancies.

## Data Availability

The original contributions presented in the study are included in the article/supplementary material, further inquiries can be directed to the corresponding author.
